# Is ultra-early endoscopy always beneficial? A systematic review and meta-analysis of timing-dependent outcomes in nonvariceal upper gastrointestinal bleeding

**DOI:** 10.7717/peerj.21702

**Published:** 2026-09-23

**Authors:** Cai-Juan Liu, Lu-Xi Xiong, Xiang-Yu Zhang, Wei-Wei Yang, Chun-Hua Liu, Logen Liu, Guo-Qing Li

**Affiliations:** 1 Department of Gastroenterology, The Second Affiliated Hospital, Hengyang Medical School, University of South China, Hengyang, China; 2 Hunan Provincial Key Laboratory of Basic and Clinical Pharmacological Research on Gastrointestinal Tumors, University of South China, Hengyang, China; 3 Clinical Research Center, The Second Affiliated Hospital, Hengyang Medical School, University of South China, Hengyang, China

**Keywords:** Nonvariceal upper gastrointestinal bleeding, Ultra-early, Endoscopy

## Abstract

**Background:**

Nonvariceal upper gastrointestinal bleeding (NVUGIB) is a common emergency with substantial morbidity and mortality. Guidelines recommend endoscopy within 24 hours, but the benefit of ultra-early endoscopy (≤6 hours) remains unclear, particularly in high-risk patients. This study aimed to systematically evaluate whether ultra-early endoscopy (≤6 hours) improves clinical outcomes compared with early endoscopy (>6 hours) in adult patients with nonvariceal upper gastrointestinal bleeding, including mortality, rebleeding, healthcare utilization, and outcomes in high-risk subgroups.

**Methods:**

A systematic review and meta-analysis of studies comparing ultra-early and early endoscopy in adults with NVUGIB was conducted. The PubMed, Embase, and Web of Science databases were searched up to January 2026 for relevant studies. Ultra-early endoscopy was defined as endoscopy performed within 6 hours of presentation, admission, or clinical diagnosis, and high-risk patients were defined according to study-level criteria, most commonly a Glasgow–Blatchford score >12 or Rockall Score >5. Pooled odds ratios (ORs) or mean differences (MDs) with 95% confidence intervals (CIs) were calculated using random-effects models, and prespecified subgroup and sensitivity analyses were used to assess the robustness of the findings.

**Results:**

Nine studies, comprising 10,785 patients (2,638 ultra-early, 8,147 early), were included in the meta-analysis, and their data analyzed. The findings suggest that ultra-early endoscopy did not significantly reduce mortality (OR 0.88, 95% CI [0.58–1.34]) or rebleeding (OR 1.23, 95% CI [0.90–1.67]), nor affect transfusion, surgical intervention, repeat endoscopy, or the length of hospital stay of the patients. However, intensive care unit (ICU) admission was more frequent in the ultra-early group (OR 1.41, 95% CI [1.20–1.66]). These findings were also consistent for patients in high-risk subgroups.

**Conclusion:**

Among adults with NVUGIB, ultra-early endoscopy provides no clear advantage over early endoscopy in terms of the key clinical outcomes, although the rate of ICU admission is higher. This suggests that management should focus on providing adequate resuscitation and timely endoscopy within an appropriate therapeutic window. Further prospective studies are warranted to evaluate the potential benefit of ultra-early endoscopy in selected high-risk patients.

## Introduction

Nonvariceal upper gastrointestinal bleeding (NVUGIB) is one of the most common gastrointestinal emergencies encountered in clinical practice and represents a leading indication for urgent endoscopic evaluation (Kanjee et al., 2021; Mega et al., 2025). NVUGIB accounts for the majority of acute upper gastrointestinal bleeding episodes, which continue to be associated with significant morbidity and mortality worldwide (Barkun et al., 2025). Estimates from large population-based studies indicate that the rate of in-hospital mortality among patients with NVUGIB generally ranges from approximately 5%–10%, with a markedly higher fatality rate observed in older patients and those with significant comorbidity burdens (Cagir, Durak & Yuksel, 2024; Jaan et al., 2025).

Despite substantial improvements in adjunctive medical treatments, including proton pump inhibitors, refinements in endoscopic hemostasis techniques, and the ongoing optimization of overall peri-emergency care pathways, the clinical impact of NVUGIB remains considerable, often requiring hospital admission, blood transfusions, and intensive healthcare resource utilization (Laine et al., 2021; Lau et al., 2023; Rees et al., 2021). These observations underscore the continued importance of optimizing management strategies for NVUGIB to improve patient outcomes and reduce the burden to healthcare systems (Asotibe et al., 2021; Elfert et al., 2023; Matsuhashi et al., 2021).

Endoscopy plays a central role in the diagnosis, risk stratification, and therapeutic intervention of NVUGIB (Martino et al., 2025; Shin et al., 2025). Current international guidelines recommend performing endoscopy within 24 h of patient stabilization to prevent unnecessary delays while allowing adequate resuscitation (Barkun et al., 2010; Gralnek et al., 2021). However, this 24-hour window is relatively broad and does not address whether earlier intervention could confer additional clinical benefits (Bai et al., 2023). With improvements in emergency endoscopy accessibility and 24-hour service availability, some centers have explored advancing the timing of endoscopy, particularly performing procedures within 6 h of presentation or diagnosis, considered ultra-early endoscopy (Stasik et al., 2025; Tejedor-Tejada et al., 2025).

The theoretical rationale for ultra-early endoscopy is that the prompt identification and management of high-risk bleeding lesions may reduce ongoing bleeding, rebleeding, and associated complications (Freitas et al., 2022). Nevertheless, studies to date evaluating this strategy have reported inconsistent results: some suggest potential reductions in mortality or rebleeding, whereas others have found no clear benefit and, in some cases, even increased intensive care unit (ICU) admission or healthcare resource utilization (Capela et al., 2024; Chang et al., 2025). Such discrepancies may reflect differences in the baseline patient risk, the adequacy of resuscitation, study design, and residual confounding issues in different studies. Additionally, the definition of “ultra-early endoscopy” varies across studies, ranging from strict 6-hour cutoffs to broader early time intervals, complicating evidence synthesis and interpretation. Additionally, evidence in high-risk patient subgroups remains limited and inconclusive, preventing firm guidance being developed on the routine application of ultra-early endoscopy (Alexandrino et al., 2019).

Given these uncertainties, an updated systematic review and meta-analysis focused specifically on the ≤6-hour threshold in adults with NVUGIB is warranted. We therefore compared ultra-early endoscopy (≤6 h) with early endoscopy (>6 h) and evaluated mortality, rebleeding, healthcare utilization, and outcomes in high-risk subgroups. This synthesis aimed to clarify whether routine acceleration of endoscopy provides additional clinical value beyond guideline-concordant early endoscopy after appropriate stabilization.

## Materials and Methods

This systematic review and meta-analysis was conducted in accordance with the Cochrane Handbook for Systematic Reviews of Interventions and is reported following the Preferred Reporting Items for Systematic Reviews and Meta-Analyses (PRISMA) statement (Page et al., 2021a; Page et al., 2021b). The study protocol was prospectively registered in the PROSPERO database (CRD420251273617) prior to initiation of the study.

### Literature search

A systematic literature search was performed in the PubMed, Embase, and Web of Science databases from their inception to January 1, 2026, to identify studies comparing ultra-early and early endoscopy in patients with NVUGIB. The search strategy incorporated both Medical Subject Headings and free-text terms related to NVUGIB, gastrointestinal endoscopy, and the timing of intervention. Core search terms included (“nonvariceal upper gastrointestinal bleeding” or “NVUGIB”) AND (“Endoscopy, Gastrointestinal” or endoscop* or EGD) AND (“Time Factors” or timing or early or “≤6” or “6-hour” or “within 6 hour”). No restrictions were applied with respect to language or publication status. The reference lists of relevant articles and previous systematic reviews were manually screened to identify additional eligible studies. The full search strategy is outlined in Table S1. The search strategy was designed and implemented independently by Cai-Juan Liu and Logen Liu. Any disagreements were resolved through discussion; if consensus could not be reached, Guo-Qing Li acted as the referee and made the final decision.

### Inclusion criteria

Studies were considered eligible if they enrolled adult patients (≥18 years old) with NVUGIB and directly compared ultra-early endoscopy with early endoscopy using extractable outcome data for the timing groups. “Ultra-early endoscopy” was defined as upper gastrointestinal endoscopy performed within 6 h of patient presentation, hospital admission, or clinical diagnosis, while “early endoscopy” referred to procedures performed more than 6 h after these time points, including specific intervals such as 6–24 h or broader comparator windows beyond 6 h when the >6-hour group was clearly defined. High-risk status, when reported, was defined by study-level criteria, typically a Glasgow-Blatchford bleeding score (GBS) >12 or Rockall Score >5. Both diagnostic and therapeutic endoscopic procedures were considered. Eligible study designs included randomized controlled trials and prospective or retrospective observational cohort studies reporting at least one relevant clinical outcome.

### Exclusion criteria

Studies were excluded if they exclusively involved patients with esophageal or gastric variceal bleeding, included pediatric populations without separable adult data, or were designed as case reports, case series, reviews, editorials, commentaries, or conference abstracts lacking sufficient extractable data. Studies without a direct comparison between ultra-early and early endoscopy were also excluded. In addition, studies reporting mixed variceal and nonvariceal bleeding were excluded if outcome data for NVUGIB could not be independently extracted according to the prespecified timing groups.

### Study selection and data extraction

Study selection and data extraction were independently conducted by two investigators using a predefined standardized data extraction form. Study titles and abstracts were initially screened to exclude clearly irrelevant studies, followed by a full-text review of the potentially eligible articles. The extracted data included study characteristics, patient demographics and baseline risk stratification (*e.g.*, Rockall or GBS scores), definitions and timing of endoscopic interventions, outcome measures of interest, and, when available, adjusted effect estimates and variables included in the adjustment models. The outcomes of interest included mortality, rebleeding, transfusion requirements, length of hospital stay, ICU admission, and need for surgical or repeat endoscopic intervention. Disagreements were resolved through discussion or consultation with a third investigator.

### Quality assessment

The methodological quality and risk of bias of the included studies were independently assessed by two reviewers. Randomized controlled trials were evaluated using the Cochrane Risk of Bias tool version 2 (Sterne et al., 2019), and non-randomized studies were assessed using the Risk Of Bias in Non-randomized Studies of Interventions (ROBINS-I) tool (Sterne et al., 2016). Any disagreements were resolved by consensus or by adjudication by a third reviewer.

### Statistical analysis

A qualitative synthesis of the included studies was initially performed. Meta-analysis was conducted when sufficient clinical and methodological homogeneity was present. For dichotomous outcomes, pooled odds ratios (ORs) with 95% confidence intervals (CIs) were calculated, while continuous outcomes were synthesized using mean differences (MDs). Statistical heterogeneity was assessed using Cochran’s Q test and quantified with the I^2^ statistic, with I^2^ values of 0–25%, 26%–50%, and >50% interpreted as indicating low, moderate, and substantial heterogeneity, respectively (Higgins & Thompson, 2002). Given the anticipated clinical heterogeneity in study design, comparator timing windows, baseline patient risk, and outcome definitions, random-effects models were used as the main analytical approach for all pooled outcomes.

Prespecified subgroup analyses were performed according to comparator timing window, baseline patient risk, and mortality outcome type. Sensitivity analyses were conducted by sequentially excluding individual studies to evaluate the robustness of the pooled estimates. Adjusted estimates were preferentially described when reported by individual studies; however, pooled analyses were primarily based on extractable event counts because adjusted effect estimates were not consistently available across studies and outcomes. Publication bias was assessed using funnel plots and Egger’s regression test (Afonso et al., 2024; Lin & Chu, 2018). All the statistical analyses were performed using Review Manager (RevMan) version 5.4 and Stata version 14.0, with two-sided *P* values <0.05 considered statistically significant.

## Results

### Basic characteristics and quality assessment

**Figure 1:**
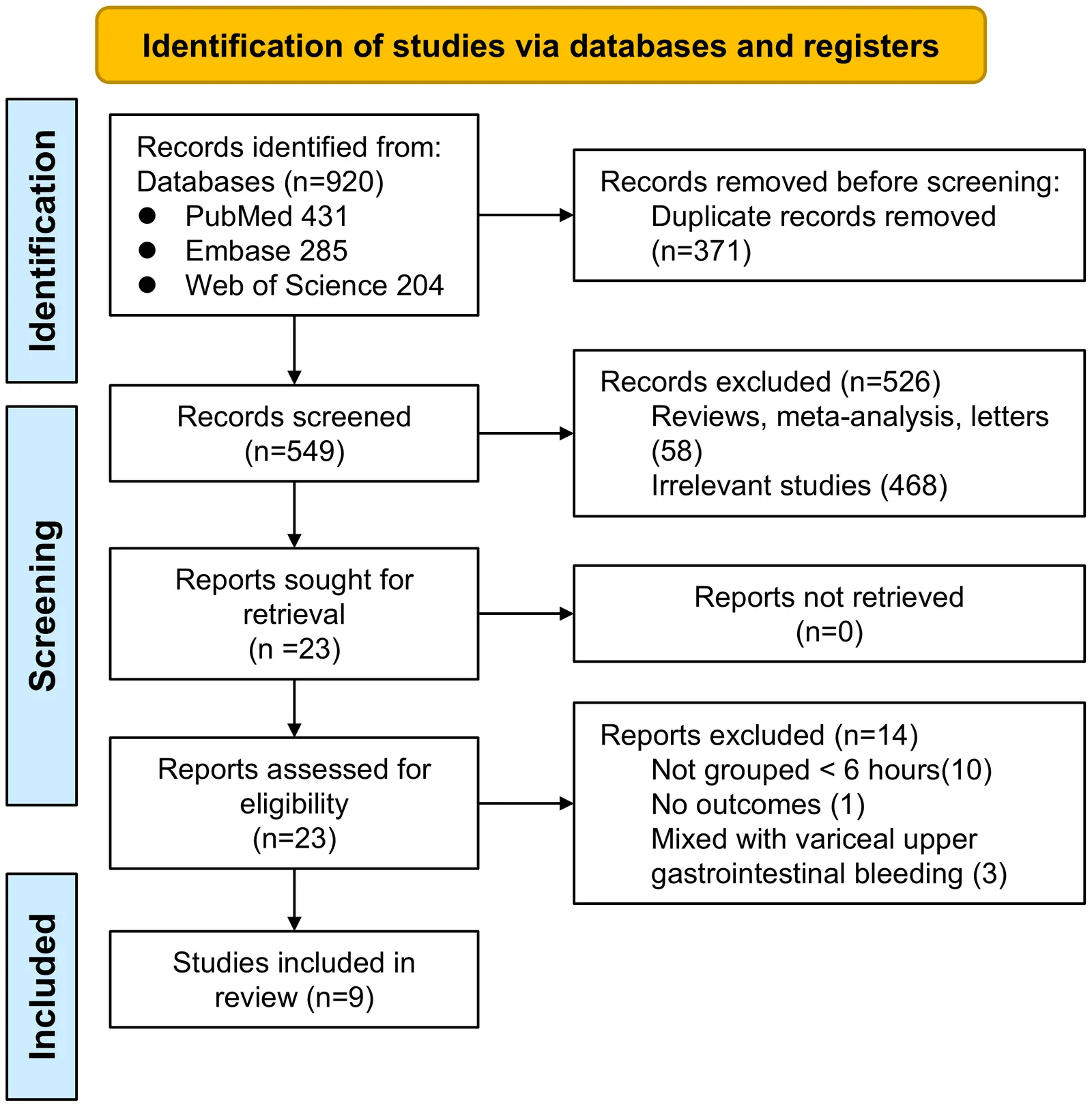
PRISMA flowchart presenting the study selection process for this systematic review and meta-analysis.

The systematic search identified 920 records from PubMed, Embase, and Web of Science with potential for consideration. After the removal of 371 duplicate records, 549 studies were screened based on their titles and abstracts, of which 526 were excluded as irrelevant or involving an inappropriate study type. This left 23 potentially eligible articles, for which the full texts were retrieved to assess their eligibility. Following this full-text evaluation, 14 studies were excluded, mainly because the endoscopy timing was not stratified using a 6-hour cutoff, relevant outcomes were not reported, or the study population included mixed variceal and nonvariceal bleeding without separable data. Ultimately, nine studies met the inclusion criteria and were included in the systematic review and meta-analysis (Bjorkman et al., 2004; Cho et al., 2018; Eid, Elliott & Rockey, 2025; Guo et al., 2022; Horibe et al., 2021; Jeon et al., 2024; Lucas Ramos et al., 2023; Sarin, Monga & Adams, 2009; Targownik et al., 2007). The study selection process is illustrated in the PRISMA flow diagram in Fig. 1.

The characteristics of the included studies are summarized in Table 1. The nine studies were published between 2004 and 2025 and were conducted in the United States, Korea, and Canada (two studies each), as well as China, Japan, and Spain (one study each). Both randomized controlled trials and observational cohort studies were included. Seven studies compared ultra-early endoscopy (≤ 6 h) with endoscopy performed within 6–24 h (Eid, Elliott & Rockey, 2025; Guo et al., 2022; Horibe et al., 2021; Jeon et al., 2024; Lucas Ramos et al., 2023; Sarin, Monga & Adams, 2009; Targownik et al., 2007), while two studies used a broader comparison window of >6 h (6–48 h) (Bjorkman et al., 2004; Cho et al., 2018). In total, 10,785 patients with NVUGIB were included, with 2,638 patients in the ultra-early endoscopy group and 8,147 in the early endoscopy group.

Baseline risk stratification was reported using either the GBS or the Rockall score. Three studies provided separate data for high-risk patients (Eid, Elliott & Rockey, 2025; Jeon et al., 2024; Lucas Ramos et al., 2023), including two studies defining high risk as a GBS greater than 12 (Jeon et al., 2024; Lucas Ramos et al., 2023) and one study using a Rockall score greater than 5 (Eid, Elliott & Rockey, 2025). Seven studies reported the number of deaths (Bjorkman et al., 2004; Cho et al., 2018; Eid, Elliott & Rockey, 2025; Horibe et al., 2021; Jeon et al., 2024; Lucas Ramos et al., 2023; Targownik et al., 2007); two studies did not report specific numbers of deaths (Guo et al., 2022; Sarin, Monga & Adams, 2009), and one study reported there were no deaths in either group (Bjorkman et al., 2004). Overall, 90 in-hospital or 30-day deaths occurred in the ultra-early endoscopy group and 111 in the early endoscopy group, corresponding to crude mortality rates of 3.67% and 5.73%, respectively.

### Quality assessment

Using ROBINS-I, seven non-randomized studies were judged as having a moderate overall risk of bias and one as having a serious overall risk of bias (Table 2), mainly because of residual confounding and selection bias inherent to observational timing studies. The RCT by Bjorkman et al. (2004) was assessed using the Cochrane Risk of Bias tool (RoB 2.0) and was rated as low risk in the domains of the randomization process, missing outcome data, and outcome measurement, with some concerns regarding deviations from intended interventions and selective reporting.

**Table 1:** Characteristics of the included studies.

**Study**	**Country**	**Design**	**Population**	**GBS**	**Rockall score**	**Definition of ultra-early endoscopy**	**Control group endoscopy**	**Total sample size (n)**	**Ultra-early group (n)**	**Control group (n)**	**Mean age (years)**	**Male (%)**	**Mortality (n)**
Guo et al. (2022)	China	RCS	All NVUGIB	8.36	NR	≤6 h	6–24 h	6,188	NR	NR	NR	NR	NR
Bjorkman et al. (2004)	USA	RCT	All NVUGIB	NR	1.7	<6 h	6–48 h	93	47	46	54.5	66.7	0 *vs* 0
Eid, Elliott & Rockey (2025) ^a^	USA	RCS	All NVUGIB	NR	NR	<6 h	6–24 h	539	105	434	53.0	65.0	11 *vs* 31
Eid, Elliott & Rockey (2025) ^b^	USA	RCS	Rockall score >5	NR	>5	<6 h	6–24 h	33	5	28	NR	NR	3 *vs* 6
Lucas Ramos et al. (2023) ^a^	Spain	POC	All NVUGIB	9.13	2.71	<6 h	6–24 h	1,096	682	414	67.0	67.4	34 *vs* 32
Lucas Ramos et al. (2023) ^b^	Spain	POC	GBS ≥12	≥12	NR	<6 h	6–24 h	403	239	164	NR	NR	24 *vs* 27
Targownik et al. (2007)	Canada	RCS	All NVUGIB	NR	3.2	≤6 h	6–24 h	169	77	92	63.0	53.3	6 *vs* 6
Horibe et al. (2021)	Japan	RCS	All NVUGIB	NR	NR	≤6 h	6–24 h	743	640	103	NR	NR	16 *vs* 7
Sarin, Monga & Adams (2009)	Canada	RCS	All NVUGIB	NR	0–3	<6 h	6–24 h	211	49	162	NR	NR	NR
Cho et al. (2018)	Korea	RCS	All NVUGIB	12.1	5.5	<6 h	6–48 h	961	571	390	57.0	81.0	9 *vs* 15
Jeon et al. (2024) ^a^	Korea	POC	All NVUGIB	39.7% >12	NR	<6 h	6–24 h	785	327	458	63.6	73.6	14 *vs* 20
Lucas Ramos et al. (2023) ^b^	Korea	POC	GBS >12	GBS >12	NR	<6 h	>6 h	616	161	455	NR	NR	NR

**Notes.**

RCS Retrospective cohort study RCT Randomized controlled trial POC Prospective observational cohort GBS Glasgow–Blatchford score NVUGIB Nonvariceal upper gastrointestinal bleeding NR Not reported h hour

a The superscript *a* indicates the overall NVUGIB population.

b The superscript *b* indicates the high-risk NVUGIB subgroup population.

**Table 2:** Risk-of-bias and methodological quality assessment of the included studies.

A. Non-randomized studies assessed using ROBINS-I
**Study**	**D1**	**D2**	**D3**	**D4**	**D5**	**D6**	**D7**	**Overall**
Guo et al. (2022)	M	M	L	M	L	L	M	M
Eid, Elliott & Rockey (2025)	M	M	L	M	L	L	M	M
Lucas Ramos et al. (2023)	M	M	L	M	L	L	M	M
Targownik et al. (2007)	S	S	L	S	L	L	M	S
Horibe et al. (2021)	M	M	L	M	L	L	M	M
Sarin, Monga & Adams (2009)	M	M	L	M	L	L	M	M
Cho et al. (2018)	M	M	L	M	L	L	M	M
Jeon et al. (2024)	M	M	L	M	L	L	M	M

B. Randomized controlled trial (Cochrane RoB 2.0)
**Study**	**D1**	**D2**	**D3**	**D4**	**D5**	**Overall**
Bjorkman et al. (2004)	Low	Some concerns	Low	Low	Some concerns	Some concerns

**Notes.**

For ROBINS-I, D1, confounding; D2, selection of participants; D3, classification of interventions; D4, deviations from intended interventions; D5, missing data; D6, measurement of outcomes; D7, selection of reported results; L, low; M, moderate; S, serious.

For RoB 2.0, D1, randomization process; D2, deviations from intended interventions; D3, missing outcome data; D4, measurement of the outcome; D5, selection of reported results; RCT, randomized controlled trial.

### Mortality

**Figure 2:**
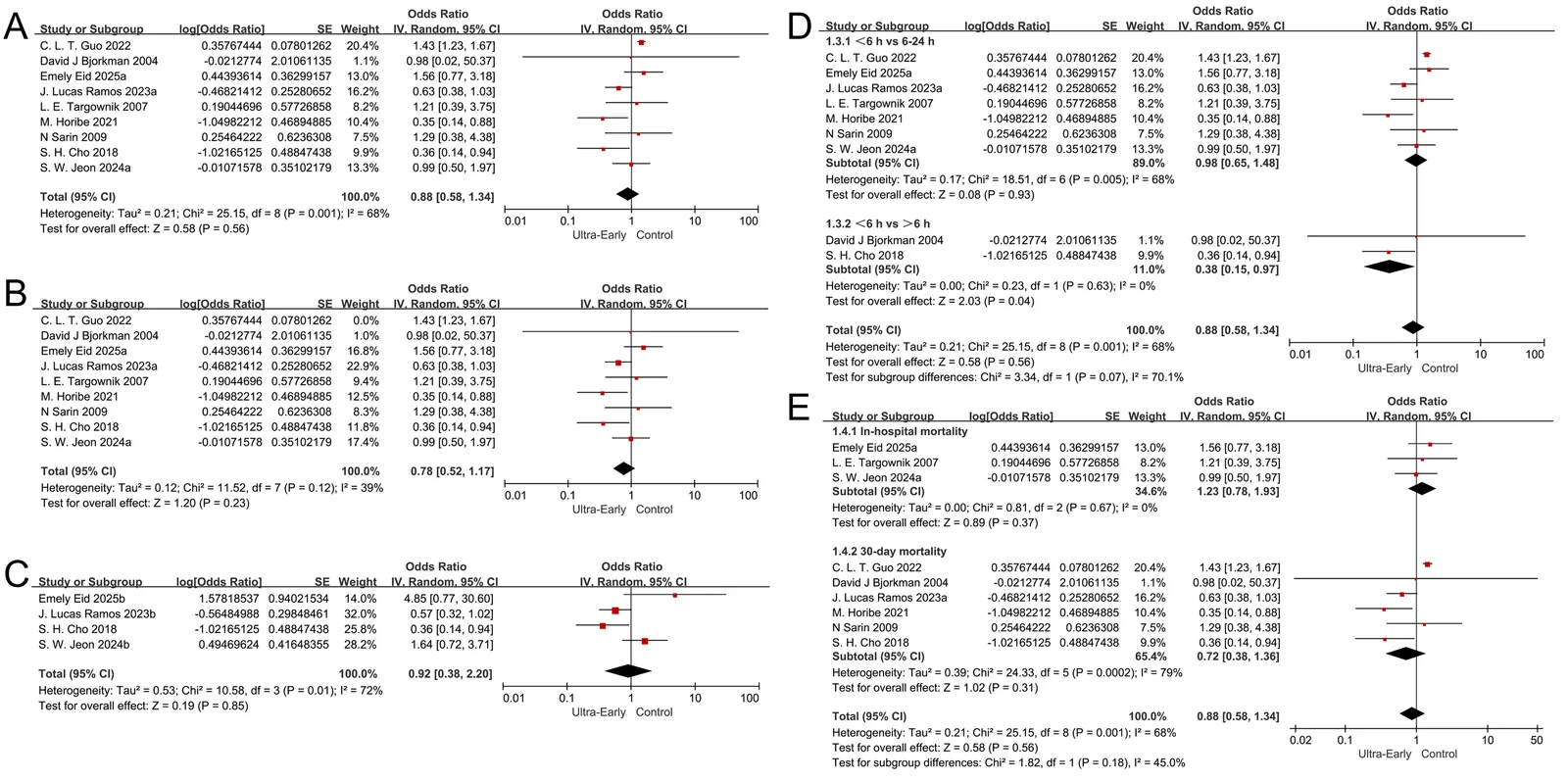
Forest plots of mortality in patients with NVUGIB. (A) Overall mortality; (B) sensitivity analysis excluding the study by Guo et al. (2022); (C) analysis among high-risk patients; (D) subgroup analysis according to endoscopy timing; (E) subgroup analysis according to the type of mortality outcome.

A total of nine studies were included to compare the mortality rate between the ultra-early group and the control group in patients with NVUGIB (Bjorkman et al., 2004; Cho et al., 2018; Eid, Elliott & Rockey, 2025; Guo et al., 2022; Horibe et al., 2021; Jeon et al., 2024; Lucas Ramos et al., 2023; Sarin, Monga & Adams, 2009; Targownik et al., 2007). Using a random-effects model, no significant difference in overall mortality was observed between the ultra-early group and the control group (OR = 0.88, 95% CI [0.58–1.34]; *P* = 0.56; Fig. 2A), with substantial heterogeneity (I^2^ = 68%).

Sensitivity analysis excluding the study by Guo et al. (2022), which had the greatest weight, shifted the pooled estimate toward reduced mortality in the ultra-early group (OR = 0.78, 95% CI [0.52–1.17]; Fig. 2B), but this remained non-significant (*P* = 0.23), with heterogeneity decreasing to I^2^ = 39%.

Among the high-risk patients, no significant difference in mortality was detected between the ultra-early group and the control group (pooled OR = 0.92, 95% CI [0.38–2.20]; *P* = 0.85; Fig. 2C), with substantial heterogeneity (I^2^ = 72%).

#### Subgroup analyses of mortality

Subgroup analyses were conducted according to endoscopy timing in the control group and the type of mortality outcome.

Regarding endoscopy timing in the control group, seven studies compared the ultra-early group with the control group undergoing endoscopy within 6–24 h (Eid, Elliott & Rockey, 2025; Guo et al., 2022; Horibe et al., 2021; Jeon et al., 2024; Lucas Ramos et al., 2023; Sarin, Monga & Adams, 2009; Targownik et al., 2007). The pooled analysis showed no significant difference in mortality between the groups (OR = 0.98, 95% CI [0.65–1.48]; *P* = 0.93; Fig. 2D), with substantial heterogeneity (I^2^ = 68%). In contrast, two studies compared the ultra-early group with a >6 h group (Bjorkman et al., 2004; Cho et al., 2018). In this subgroup analysis, the ultra-early group was found to be associated with a lower mortality risk (OR = 0.38, 95% CI [0.15–0.97]; *P* = 0.04), with low heterogeneity (I^2^ = 0%). The test for subgroup differences did not reach statistical significance (*P* = 0.07).

Regarding the mortality outcome type, three studies reported in-hospital mortality (Eid, Elliott & Rockey, 2025; Jeon et al., 2024; Targownik et al., 2007). The pooled analysis showed no significant difference in in-hospital mortality between the ultra-early group and the control group (OR = 1.23, 95% CI [0.78–1.93]; *P* = 0.37; Fig. 2E), with low heterogeneity (I^2^ = 0%). In contrast, six studies reported 30-day mortality (Bjorkman et al., 2004; Cho et al., 2018; Guo et al., 2022; Horibe et al., 2021; Lucas Ramos et al., 2023; Sarin, Monga & Adams, 2009). The pooled estimate similarly demonstrated no significant reduction in 30-day mortality in the ultra-early group compared with the control group (OR = 0.72, 95% CI [0.38–1.36]; *P* = 0.31), with substantial heterogeneity (I^2^ = 79%). The test for subgroup differences between in-hospital mortality and 30-day mortality did not reach statistical significance (*P* = 0.18).

### Rebleeding

**Figure 3:**
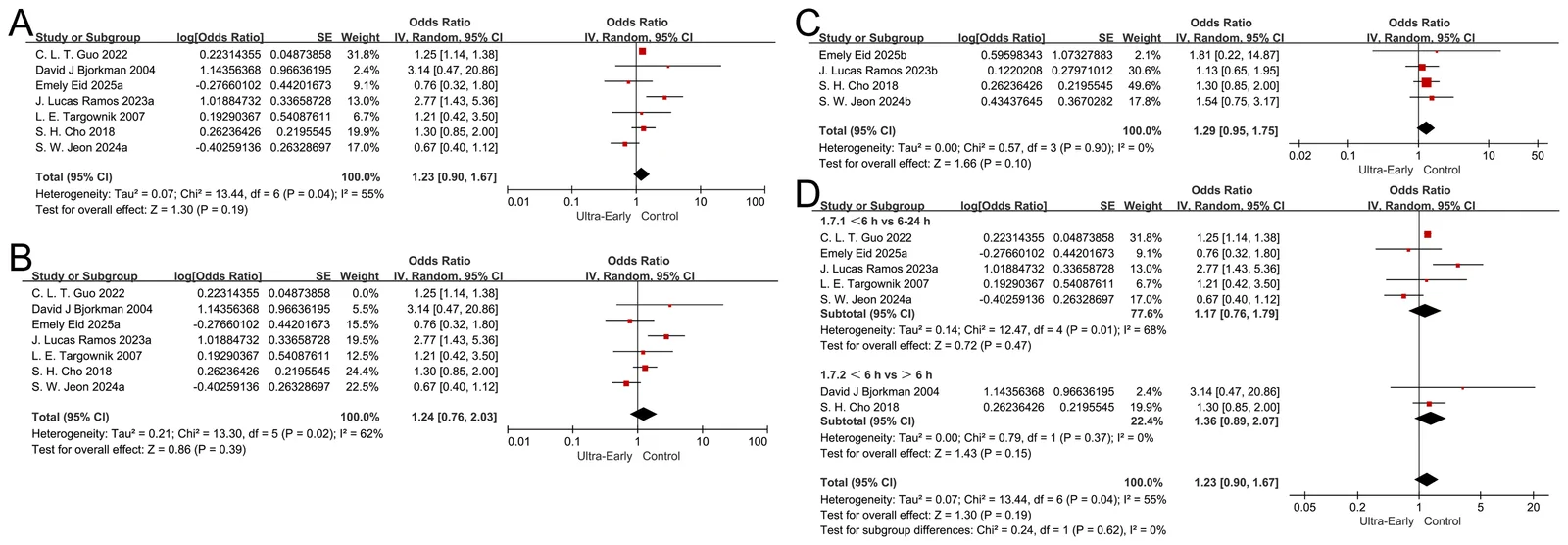
Forest plots of rebleeding in patients with NVUGIB. (A) Overall rebleeding; (B) sensitivity analysis excluding the study by Guo et al. (2022); (C) analysis among high-risk patients; (D) subgroup analysis according to endoscopy timing.

A total of seven studies compared rebleeding between the ultra-early group and the control group in patients with NVUGIB (Bjorkman et al., 2004; Cho et al., 2018; Eid, Elliott & Rockey, 2025; Guo et al., 2022; Jeon et al., 2024; Lucas Ramos et al., 2023; Targownik et al., 2007). Using a random-effects model, ultra-early endoscopy was not associated with a significant change in rebleeding risk compared with the control group (OR = 1.23, 95% CI [0.90–1.67]; *P* = 0.19; Fig. 3A), with substantial heterogeneity (I^2^ = 55%).

Sensitivity analysis excluding the study with the largest weight (31.8%) (Guo et al., 2022), showed a similar non-significant effect on rebleeding (OR = 1.24, 95% CI [0.76–2.03], *P* = 0.39; Fig. 3B), with heterogeneity increasing to I^2^ = 62%.

Among high-risk patients, no significant difference in rebleeding was detected between the two groups (pooled OR = 1.29, 95% CI [0.95–1.75]; *P* = 0.10; Fig. 3C), with low heterogeneity (I^2^ = 0%).

#### Subgroup analyses of rebleeding

Subgroup analyses were performed according to endoscopy timing in the control group. Five studies compared the ultra-early group with the control group undergoing endoscopy within 6–24 h (Eid, Elliott & Rockey, 2025; Guo et al., 2022; Jeon et al., 2024; Lucas Ramos et al., 2023; Targownik et al., 2007), and the pooled analysis showed no significant difference in rebleeding between the groups (OR = 1.17, 95% CI [0.76–1.79]; *P* = 0.47; Fig. 3D), with substantial heterogeneity (I^2^ = 68%). Two studies compared the ultra-early group with the >6 h group (Bjorkman et al., 2004; Cho et al., 2018), and the pooled estimate likewise found no significant difference in rebleeding (OR = 1.36, 95% CI [0.89–2.07]; *P* = 0.15), with low heterogeneity (I^2^ = 0%). The test for subgroup differences did not show a statistically significant difference between the subgroups (*P* = 0.62).

### Additional outcomes

In the present study, we further evaluated the impact of ultra-early endoscopy on secondary outcomes, including transfusion requirement, transfusion units, number of transfusion units, ICU admission, need for intervention, surgery, and length of hospital stay.

#### Transfusion requirement

**Figure 4:**
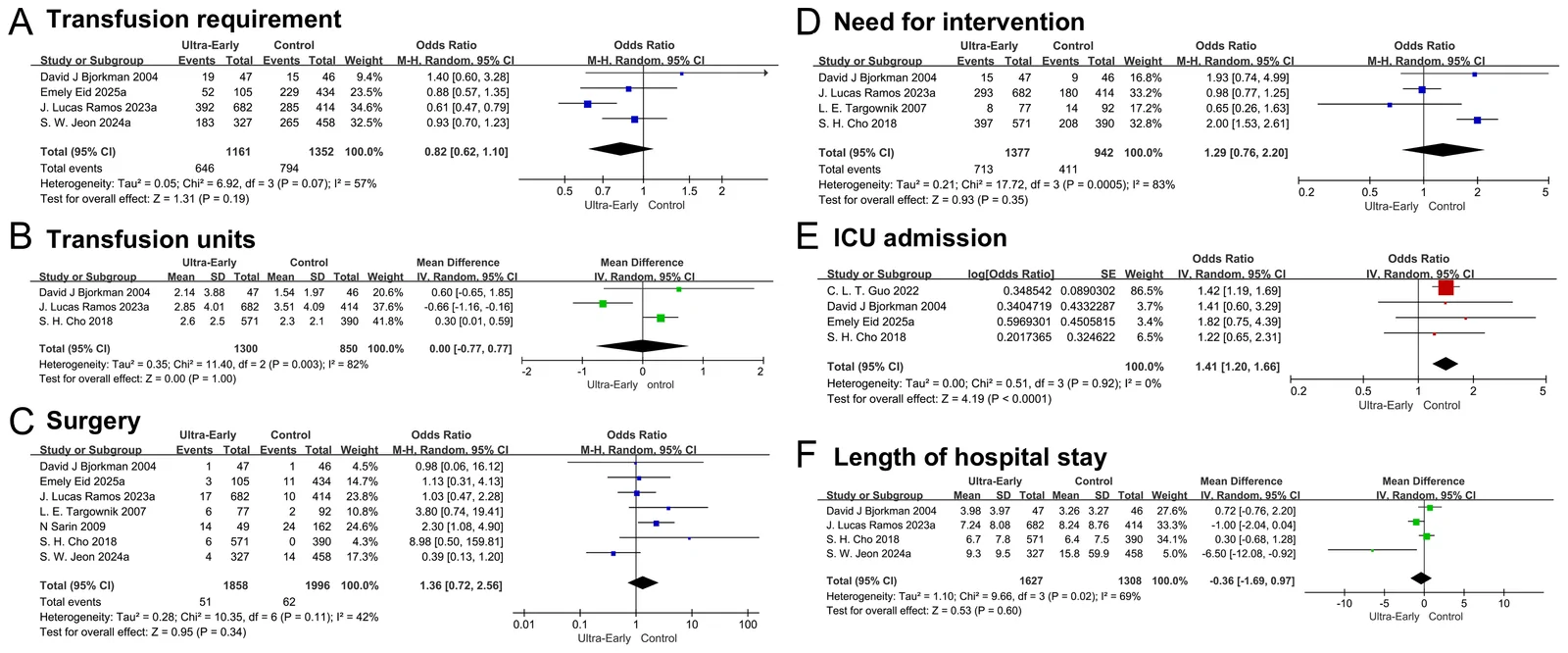
Forest plots for additional clinical outcomes in patients with NVUGIB. (A) Transfusion requirement; (B) number of transfusion units; (C) surgical intervention; (D) need for intervention; (E) ICU admission; (F) length of hospital stay.

Four studies reported transfusion requirements among patients (Bjorkman et al., 2004; Eid, Elliott & Rockey, 2025; Jeon et al., 2024; Lucas Ramos et al., 2023). Pooled analysis using a random-effects model showed that ultra-early endoscopy did not significantly reduce the transfusion requirement (OR = 0.82, 95% CI [0.62–1.10]; *P* = 0.19; Fig. 4A), with substantial heterogeneity (I^2^ = 57%).

#### Transfusion units

Three studies reported the number of transfusion units (Bjorkman et al., 2004; Cho et al., 2018; Lucas Ramos et al., 2023). Random-effects meta-analysis showed there was no significant difference between the ultra-early group and the control group (MD = 0.00, 95% CI [−0.77 to 0.77]; *P* = 1.00; Fig. 4B), with substantial heterogeneity (I^2^ = 82%).

#### Surgery

Seven studies reported the surgical rates (Bjorkman et al., 2004; Cho et al., 2018; Eid, Elliott & Rockey, 2025; Jeon et al., 2024; Lucas Ramos et al., 2023; Sarin, Monga & Adams, 2009; Targownik et al., 2007). Random-effects meta-analysis showed there was no significant difference between the ultra-early group and the control group for the rate of surgery (OR = 1.36, 95% CI [0.72–2.56]; *P* = 0.34; Fig. 4C), with moderate heterogeneity (I^2^ = 42%).

#### Need for intervention

Four studies reported the need for repeat intervention (Bjorkman et al., 2004; Cho et al., 2018; Lucas Ramos et al., 2023; Targownik et al., 2007). Random-effects meta-analysis found no significant difference between the ultra-early group and the control group (OR = 1.29, 95% CI [0.76–2.20]; *P* = 0.35; Fig. 4D), with substantial heterogeneity (I^2^ = 83%).

#### ICU admission

Four studies reported ICU admission (Bjorkman et al., 2004; Cho et al., 2018; Eid, Elliott & Rockey, 2025; Guo et al., 2022). Random-effects meta-analysis indicated that the ultra-early group had a significantly higher risk of ICU admission compared with the control group (OR = 1.41, 95% CI [1.20–1.66]; *P* < 0.0001; Fig. 4E), with low heterogeneity (I^2^ = 0%).

#### Length of hospital stay

Four studies reported the length of hospital stay (Bjorkman et al., 2004; Cho et al., 2018; Jeon et al., 2024; Lucas Ramos et al., 2023). Random-effects meta-analysis showed there was no significant difference between the ultra-early group and the control group in this regard (MD = −0.36, 95% CI [−1.69 to 0.97]; *P* = 0.60; Fig. 4F), with substantial heterogeneity (I^2^ = 69%).

#### Publication bias

**Figure 5:**
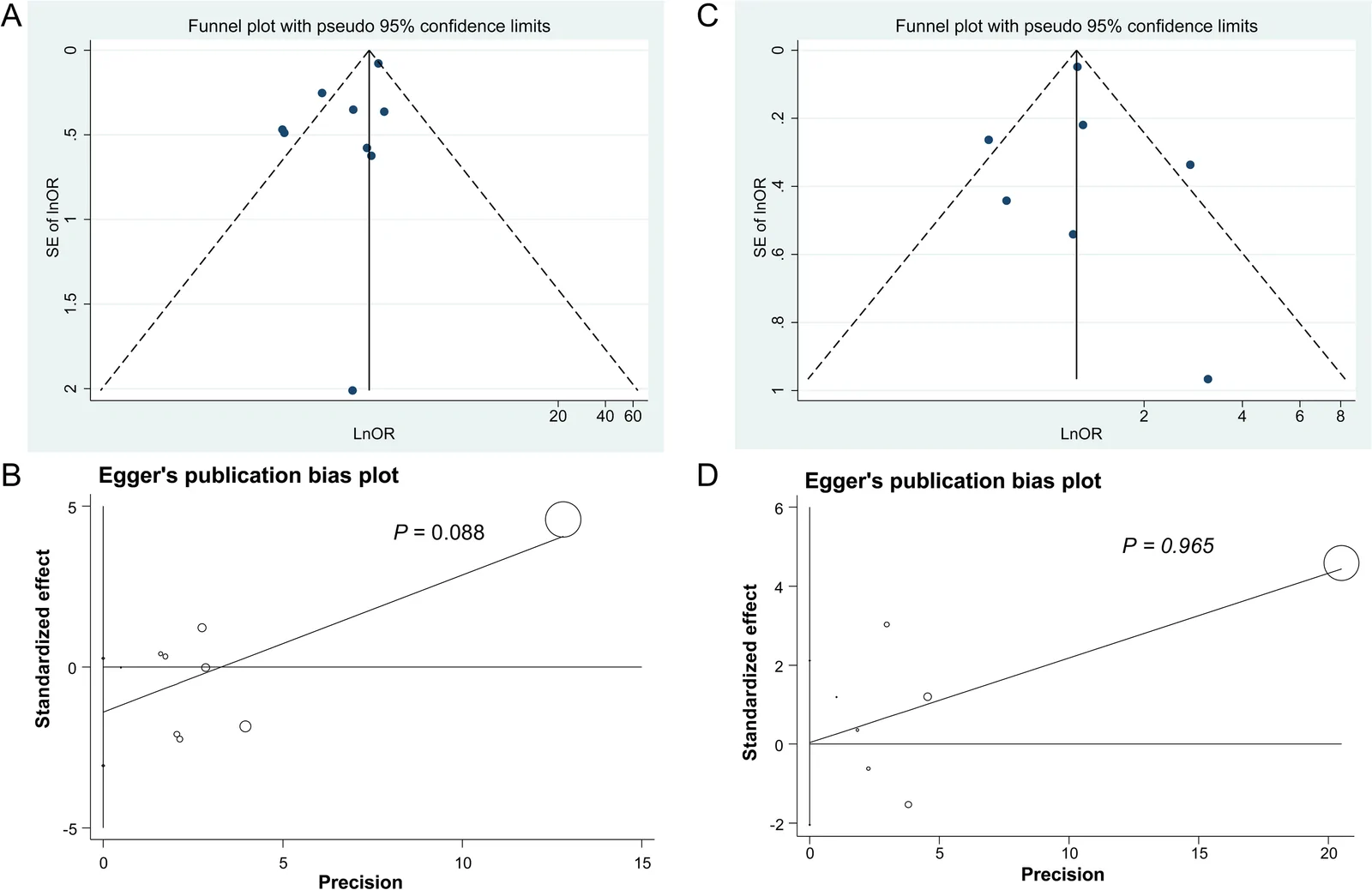
Funnel plots and Egger’s test regression plots for the assessment of publication bias. (A) Funnel plot for studies reporting mortality; (B) Egger’s test regression plot for mortality; (C) funnel plot for studies reporting rebleeding; (D) Egger’s test regression plot for rebleeding.

Publication bias was assessed using funnel plots and Egger’s test. For mortality, the funnel plot showed no obvious asymmetry, and the Egger’s test intercept was not significant (Coef. = −1.4015, 95% CI [−3.0724 to 0.2694]; *P* = 0.088; Figs. 5A, 5B). Similarly, for rebleeding, the funnel plot was symmetric, and the Egger’s test intercept was also not significant (Coef. = 0.0377, 95% CI [−2.0389 to 2.1143]; *P* = 0.965; Figs. 5C, 5D), suggesting there was no evidence of publication bias for either outcome.

## Discussion

In this systematic review and meta-analysis, we comprehensively compared ultra-early endoscopy performed within 6 h with early endoscopic strategies in adult patients presenting with NVUGIB. Across our pooled analyses, ultra-early endoscopy was not associated with significant reductions in all-cause mortality, rebleeding, transfusion requirements, need for repeat endoscopy or surgical intervention, or length of hospital stay. These findings remained largely consistent across predefined subgroups and sensitivity analyses, despite the presence of moderate heterogeneity among the included studies.

These findings are consistent with previous evidence suggesting that routine urgent or very early endoscopy does not consistently improve major outcomes compared with endoscopy within 24 h (Ahn et al., 2016; Guan et al., 2022). By focusing on NVUGIB and a strict ≤6-hour threshold, the present analysis further suggests that endoscopic timing may be governed by a “sufficiently early” threshold rather than a universal “earlier is better” principle. For most patients, completion of endoscopy within 24 h after adequate hemodynamic stabilization may already provide sufficient opportunity for lesion identification, risk stratification, and endoscopic hemostasis (Güven et al., 2023; Lomoc, Prudencio & Dillera, 2025). Other observational studies suggest that clinical outcomes may also vary according to care setting, admission timing, and healthcare-resource factors (Arslan et al., 2021; Saleem et al., 2020). A contemporary review emphasized that urgent endoscopy should be balanced against adequate stabilization rather than applied indiscriminately (Lau, 2022). Patient-level factors, including comorbidity burden and ASA or physiologic status, may have a greater influence on prognosis than timing alone (Bucci et al., 2024). Similarly, a randomized trial in high-risk acute upper gastrointestinal bleeding found no mortality benefit from endoscopy within 6 h compared with endoscopy within 6–24 h (Lau et al., 2020). Therefore, the available evidence suggests that shortening the endoscopy window to ≤6 h alone may not provide additional clinical benefit over timely endoscopy after adequate stabilization; clinical decisions should instead incorporate pre-endoscopic optimization and individualized risk assessment.

With respect to secondary outcomes, ultra-early endoscopy was found to be associated with a higher rate of ICU admission. Notably, this increase was not accompanied by improvements in mortality, rebleeding, or the need for surgical intervention. This finding can be more plausibly explained by selection bias inherent to clinical decision-making rather than a direct adverse effect of ultra-early endoscopy itself. This interpretation is consistent with large observational data showing higher ICU admission in patients undergoing urgent endoscopy without corresponding improvements in mortality or rebleeding (Guo et al., 2022). From a health system perspective, the absence of an improvement in major clinical outcomes, combined with increased ICU utilization and off-hours endoscopy staffing demands, raises questions about the potential resource implications *versus* clinical benefits of routinely adopting an ultra-early endoscopy strategy.

It is important to emphasize that, aside from increased ICU admission, current evidence does not demonstrate any clear harm associated with ultra-early endoscopy in terms of mortality, rebleeding, or surgical intervention rates. This suggests that, in centers with sufficient resources and experienced endoscopists, ultra-early endoscopy appears to be safe. Nevertheless, given the lack of a demonstrable benefit in key outcomes and the potential for increased resource consumption, existing evidence does not support the routine implementation of ultra-early endoscopy for all NVUGIB patients. Its application should therefore remain selective and individualized.

Several limitations should be acknowledged. First, most included studies were observational, and endoscopy timing was often determined by clinical judgment; therefore, residual confounding and confounding by indication cannot be excluded, even when individual studies adjusted for baseline risk. Second, clinical heterogeneity existed across studies, including differences in comparator timing windows, outcome definitions, and criteria used to define high-risk patients, which may have reduced the precision of pooled estimates. Third, only a limited number of studies reported outcomes specifically among high-risk patients, and these subgroup analyses were constrained by small sample sizes and inconsistent risk stratification methods. Therefore, the absence of a clear benefit in high-risk patients should be interpreted cautiously. Future studies should incorporate standardized timing definitions, detailed pre-endoscopic risk assessment, and prospective adjustment for resuscitation status and comorbidity burden.

Taken together, these findings should be interpreted in the context of current clinical practice and an increasing interest in expedited endoscopic strategies for NVUGIB. The effect of endoscopic timing appears to depend on the patient’s stability, bleeding severity, and overall clinical management rather than timing alone. Consequently, decisions regarding endoscopic timing should be guided by individual clinical circumstances and healthcare system considerations.

## Conclusion

In adult patients with NVUGIB, currently available evidence does not demonstrate that routine endoscopy within 6 h improves mortality, rebleeding, or other key clinical outcomes compared with early endoscopic strategies. Although ultra-early endoscopy was found to be associated with higher ICU admission rates, no clear adverse effects on major clinical outcomes were identified. Clinical management should emphasize adequate resuscitation, careful risk assessment, and timely endoscopy within an appropriate therapeutic window, rather than indiscriminately advancing the endoscopic timing. Further high-quality prospective studies are required to clarify whether ultra-early endoscopy would provide a meaningful benefit in selected patient populations.

##  Supplemental Information

10.7717/peerj.21702/supp-1 Supplemental Information 1 PRISMA checklist

10.7717/peerj.21702/supp-2 Supplemental Information 2 The search strategy of each database

10.7717/peerj.21702/supp-3 Supplemental Information 3 Raw data extracted from the cited literature Raw extracted data used in this systematic review and meta-analysis. The dataset includes all information extracted from the included studies (study characteristics and outcomes) and all data used for figure/table generation and quantitative synthesis.

10.7717/peerj.21702/supp-4 Supplemental Information 4 Intended audience

## Additional Information and Declarations

### Data Availability

The following information was supplied regarding data availability: Raw data extracted from the cited literature.
