# Supplementary material for: Is ultra-early endoscopy always beneficial? A systematic review and meta-analysis of timing-dependent outcomes in nonvariceal upper gastrointestinal bleeding
Source: PeerJ. 2026 Sep 23;14:e21702. doi: 10.7717/peerj.21702 (PMC13615735; doi:10.7717/peerj.21702)
Supplement: Supplementary file 2 [file peerj-14-21702-s002.docx]

**Supplementary Table 1** The search strategy of each database

| **Database** | **Search strategy** | **Results** |
| --- | --- | --- |
| **PubMed** | #1 "nonvariceal upper gastrointestinal bleeding"[tiab] OR "non-variceal upper gastrointestinal bleeding"[tiab] OR NVUGIB[tiab] OR (("Peptic Ulcer Hemorrhage"[Mesh] OR "peptic ulcer bleeding" OR "peptic ulcer hemorrhage"[tiab] OR “Upper Gastrointestinal Hemorrhage”[tiab] OR "upper gastrointestinal bleed*"[tiab] OR hematemesis[tiab] OR melena[tiab] OR UGIB[tiab]) AND (nonvariceal[tiab] OR non-variceal[tiab])) | 1,082 |
|  | #2 Endoscopy, Gastrointestinal[Mesh] OR endoscop*[tiab] OR gastroscop*[tiab] OR EGD[tiab] | 341,664 |
|  | #3 "Time Factors"[Mesh] OR timing[tiab] OR early[tiab] OR urgent[tiab] OR emergent[tiab] OR Ultra-Early[tiab] OR "≤6"[tiab] OR "<=6"[tiab] OR "6 h"[tiab] OR "6-hour"[tiab] OR "within 6 hour*"[tiab] | 8,118,407 |
|  | #4 #1 AND #2 AND #3 | **431** |
| **Embase** | #1 ‘nonvariceal upper gastrointestinal bleeding’:ti,ab OR ‘non-variceal upper gastrointestinal bleeding’:ti,ab OR NVUGIB:ti,ab OR ((‘Peptic Ulcer Hemorrhage’/exp OR ‘peptic ulcer bleeding’:ti,ab OR ‘peptic ulcer hemorrhage’:ti,ab OR ‘Upper Gastrointestinal Hemorrhage’:ti,ab OR ‘upper gastrointestinal bleed*’:ti,ab OR hematemesis:ti,ab OR melena:ti,ab OR UGIB:ti,ab) AND (nonvariceal OR non-variceal:ti,ab)) | 2,162 |
|  | #2 ‘Endoscopy, Gastrointestinal’/exp OR endoscop*:ti,ab OR gastroscop*:ti,ab OR EGD:ti,ab | 607,626 |
|  | #3 ‘Time Factors’/exp OR timing:ti,ab OR early:ti,ab OR urgent:ti,ab OR emergent:ti,ab OR Ultra-Early:ti,ab OR ‘≤6’:ti,ab OR ‘6 h’:ti,ab OR ‘6-hour’:ti,ab OR ‘within 6 hour*’:ti,ab | 10,693,882 |
|  | #4 #1 AND #2 AND #3 | 1,054 |
|  | #5 #1 AND #2 AND #3 AND ('article'/it OR 'article in press'/it) AND [humans]/lim AND [clinical study]/lim | **285** |
| **Web of Science** | TS=("nonvariceal upper gastrointestinal bleeding" OR "non-variceal upper gastrointestinal bleeding" OR NVUGIBOR (("Peptic Ulcer Hemorrhage" OR "peptic ulcer bleeding" OR "peptic ulcer hemorrhage" OR “Upper Gastrointestinal Hemorrhage” OR "upper gastrointestinal bleed*" OR hematemesis OR melena OR UGIB) AND (nonvariceal OR non-variceal))) AND (Endoscopy, Gastrointestinal OR endoscop* OR gastroscop* OR EGD) AND ("Time Factors" OR timing OR early OR urgent OR emergent OR Ultra-Early OR "≤6" OR "<=6" OR "6 h" OR "6-hour"OR "within 6 hour*")  Refined by: Document types: Article | **204** |
