# Supplementary material for: Is ultra-early endoscopy always beneficial? A systematic review and meta-analysis of timing-dependent outcomes in nonvariceal upper gastrointestinal bleeding
Source: PeerJ. 2026 Sep 23;14:e21702. doi: 10.7717/peerj.21702 (PMC13615735; doi:10.7717/peerj.21702)
Supplement: Supplementary file 3 [file peerj-14-21702-s004.docx]

**Intended audience**

This manuscript is intended for clinicians and researchers involved in gastroenterology and emergency medicine, particularly endoscopists and physicians managing nonvariceal upper gastrointestinal bleeding (NVUGIB). It may also be of interest to guideline developers, hospital administrators, and health services researchers concerned with optimizing endoscopy timing and resource allocation (e.g., ICU utilization).
